# Umbilical Cord Mesenchymal Stem Cells Attenuate Podocyte Injury in Diabetic Nephropathy Rats by Inhibiting Angpltl4/Integrin *β*3 in the Glomerulus

**DOI:** 10.1155/jdr/6683126

**Published:** 2025-04-11

**Authors:** Shiyuan Liu, Mingyao Meng, Chunkai Huang, Lijia He, Pu Wang, Zhe Tang, Xi Ran, Hui Gao, Yangfan Guo, Yan He, Jian Chen, Haiyan Hu, Shan He, Yiyi Zhao, Zongliu Hou, Lin Li, Wenhong Li, Wenju Wang, Xiaodan Wang

**Affiliations:** ^1^Yan'an Hospital Affiliated to Kunming Medical University, Kunming, China; ^2^Key Laboratory of Tumor Immunological Prevention and Treatment of Yunnan Province, Kunming, China; ^3^Yunnan stem cell Clinical transformation Engineering Research Center, Kunming, China

## Abstract

In this study, we investigated the therapeutic effects and mechanisms of umbilical cord mesenchymal stem cells (UCMSCs) in diabetic nephropathy (DN) ZDF (FA/FA) rats. The therapeutic effects were assessed by renal function tests, the urinary albumin–creatinine ratio, PAS staining, electron microscopy, and TGF-*β*1 expression in renal tissue. Subsequently, podocyte injury in renal tissue was detected by immunofluorescence staining for podocin. To further explore the underlying mechanism, serum Angptl4 levels were measured, and Angptl4, integrin *β*3, fibronectin, and podocin levels in renal tissue were analysed by Western blotting. In vitro, podocytes are stimulated with high glucose and then treated with UCMSCs, and podocyte activity and the expression of synaptopodin, Angptl4, and integrin *β*3 were observed. UCMSC significantly improve renal function, pathological injury, and podocyte injury in the ZDF (FA/FA) rats. Western blot revealed increased expression of Angptl4, integrin *β*3, and fibronectin in renal tissues of the DN group, and UCMSC treatment significantly downregulated those proteins. However, UCMSC showed no effects on serum Angptl4 concentration. Podocin expression in renal tissues was significantly restored by UCMSC treatment. In vitro, podocyte activity was decreased after high glucose stimulation and improved by UCMSC treatment. UCMSC restored the expression of synaptopodin, and Angptl4 and downstream integrin *β*3 were also inhibited. Our study suggested that UCMSC therapy could improve renal function and renal pathological changes in ZDF (FA/FA) rats. In addition, inhibition of the Angptl4/integrin *β*3 pathway is the potential mechanism by which UCMSC attenuates podocyte injury in the DN model.

## 1. Introduction

With changes in diet and lifestyle, the incidence of diabetes mellitus (DM) is increasing rapidly. The current global incidence of diabetes is 564 million and is expected to increase by 783 million by 2045 [1]. Diabetic nephropathy (DN) is the most serious chronic microvascular complication of diabetes and one of the main causes of death in DM patients, affecting about 40% of diabetic patients. DN eventually progresses to end-stage renal disease (ESRD), and DN is the main cause of ESRD [2]. At present, the clinical treatment of DN is very difficult, and the main treatment options include blood glucose control, blood pressure control, lifestyle management, correction of lipid metabolism disorders, dialysis treatment, and kidney transplantation. Although angiotensin-converting enzyme inhibitors, angiotensin receptor blocker, sodium–glucose Cotransporter 2 inhibitors, and nonsteroidal mineral corticosteroid receptor antagonists have been used for the treatment of DN [3], the prevention and treatment of DN are still major clinical challenges.

In recent years, mesenchymal stem cells have been used in the treatment of DN. In 2016, scholars explored the therapeutic effect of allogeneic bone marrow mesenchymal precursor cells (BMSCs) in DN patients. Compared with placebo, a single intravenous infusion of allogeneic BMSCs showed a trend toward stabilizing or improving estimated glomerular filtration rate (eGFR) and measured glomerular filtration rate (mGFR) at week 12 after infusion [4]. In 2023, the safety, tolerability, and preliminary efficacy of Orbcel-M bone marrow-derived, anti-CD362-selected allogeneic mesenchymal stem cells (Orbcel-M) in adult patients with Type 2 diabetes and progressive DN were investigated. Allogeneic MSC intervention was safe and well tolerated. Patients receiving cell therapy had a significantly lower rate of decline in eGFR (but not mGFR) at 18 months compared with placebo [5]. Those studies showed that MSC treatment delays the renal function deterioration in DN patients. However, the mechanism of MSCs in the treatment of DN is still unclear, and a large number of basic studies are needed to explore the mechanism, so as to lay a foundation for improving the therapeutic effect of DN by engineering MSCs.

An increasing studies have shown that the occurrence and development of DN were closely related to podocyte injury [6]. Podocytes are terminally differentiated epithelial cells that maintain the glomerular filtration barrier. In the pathogenesis of DN, podocyte damage, such as changes in podocyte structure or a reduction in the number of podocytes, affects the integrity of glomerular filtration barrier and leads to proteinuria, thereby accelerating the progression of DN [7–9]. Human umbilical cord-derived mesenchymal stem cells (UCMSCs) prevent the progression of early DN through inhibiting inflammation [10]. MSCs play an important role in the treatment of DN by protecting podocytes [11], but the specific underlying mechanism is not clearly stated.

Scholars have found that podocyte injury is closely related to angiopoietin-like 4 (Angptl4) [12]. There are two types of Angptl4 proteins in the body, namely, circulating Angptl4 and Angptl4, which aremsecreted by podocytes [13]. Circulating Angptl4 mainly regulates lipoprotein metabolism [14, 15]. Although the level of Angptl4 in normal kidney tissues is low, a study revealed that the expression of Angptl4 in kidney tissues was significantly increased in a DN model and that podocytes were the source of Angptl4 [16]. The secretion of Angptl4 by podocytes is closely related to DN podocyte injury. Podocyte-specific Angptl4 transgene overexpression (NPHS2-Angptl4) in rats results in a large amount of proteinuria and foot process fusion [17]. The Angptl4 secreted by podocytes in the DN is upregulated and can be detected in urine. Studies have found that the expression of Angptl4 in urine is closely related to proteinuria in DN patients. Angptl4 is a marker of podocytes injury and may become a potential target for DN treatment [16]. At present, the downstream signalling pathway of Angptl4 in podocyte injury has not been identified. The integrin family is involved in podocyte damage and relies on integrins to attach to the GBM [18]. Studies have shown that integrin *β*3 plays a key role in podocyte injury [19]. Integrin *β*3 is involved in podocyte movement, and activation of integrin *β*3 leads to loss of the foot process and proteinuria by activating downstream intracellular signalling molecules [20]. The interaction between Angptl4 and integrin *β*3 has been verified in a hypoxia-induced vascular permeability model [21]. However, the mechanism of Angptl4 and integrin *β*3 in podocyte injury in DN has not been reported. In this study, UCMSC were used to treat ZDF (FA/FA) rats, which have pathological lesions similar to those of DN in humans [22], to further explore the mechanism by which UCMSC protect podocytes.

## 2. Materials and Methods

### 2.1. Preparation of Human Umbilical Cord Mesenchymal Stem Cells

**Figure 1:**
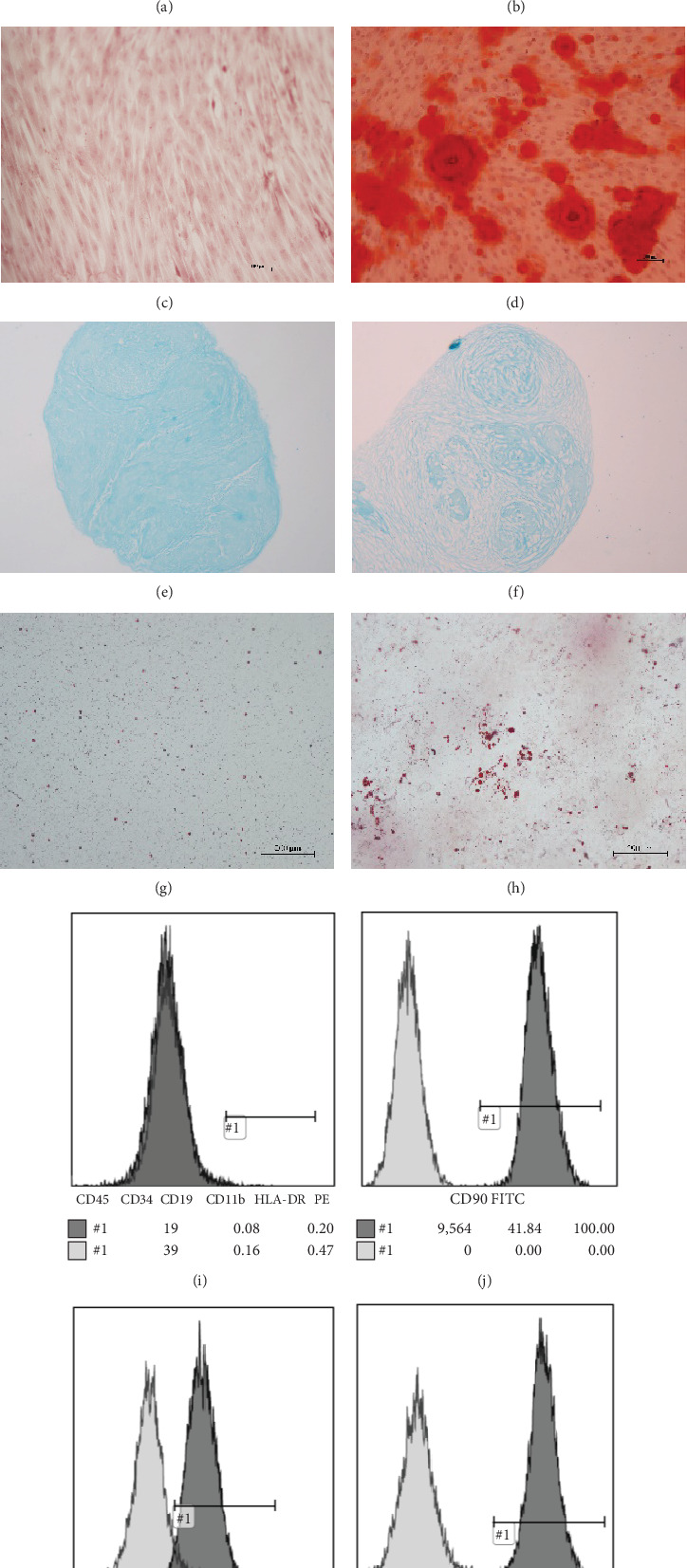
Phenotype and morphological identification of UCMSC. Mesenchymal stem cells were morphologically amplified (a) 50 times or (b) 100 times. (c, d) Alizarin Red staining confirmed the successful differentiation of UCMSC into osteoblasts, as shown in the (c) control and (d) induction images taken under a ×200 magnifying glass. (e, f) Simple blue dye confirmed the successful differentiation of UCMSC into chondrocytes. Representative photographs of the (e) control and (f) induction groups were taken under a ×100 magnification glass. (g, h) Oil Red O staining confirmed the successful differentiation of UCMSC into adipocytes, as shown in the (g) control and (h) induction images taken at ×100 magnification. The UCMSC surface markers CD90, CD105, CD73, CD45, CD34, CD11b, CD19, and HLA-DR were detected by (i–l) flow cytometry. The percentages of CD90-, CD105-, and CD73-positive cells were all above 98%, and the remaining cells were almost negative.

UCMSC were provided by the Central Laboratory of Yan'an Hospital Affiliated with Kunming Medical University. Cell phenotypes of UCMSC were detected by flow cytometric analysis. In addition, UCMSCs are induced to differentiation into adipocytes, osteoblasts, and chondrocytes [23] (Figure 1).

### 2.2. Animal Experiments

#### 2.2.1. Establishment of Animal Models and Experimental Group

**Figure 2:**
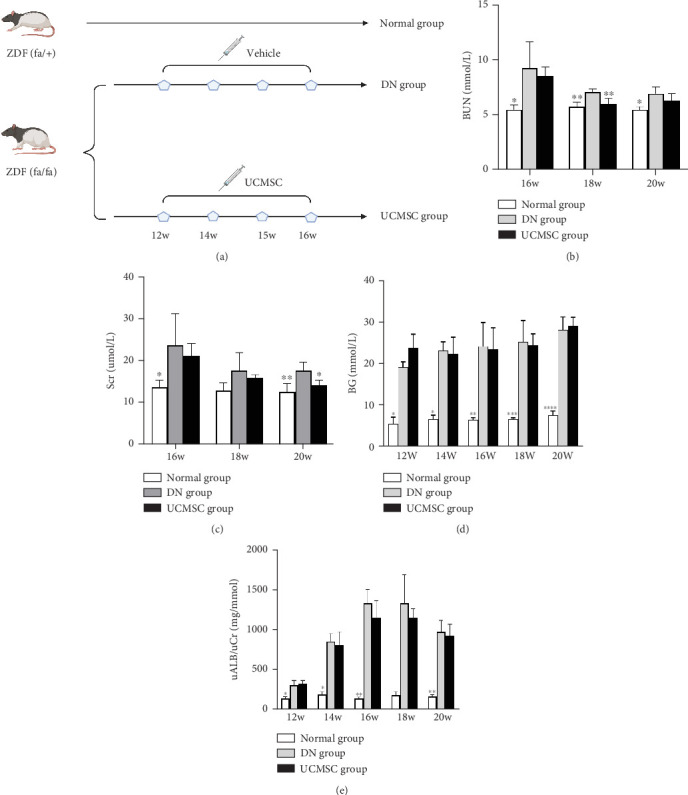
(a) Schematic representation of the animal experiments (created by BioRender). Biochemical indices of each group. (b) Blood urea nitrogen level. (c) Serum creatinine level. (d) Blood glucose level. (e) Urinary albumin–creatinine ratio. versus DN group. ∗*p* < 0.05, ∗∗*p* < 0.01, ∗∗∗*p* < 0.001, ∗∗∗∗*p* < 0.0001.

Male ZDF rats were purchased from Beijing Vi Tong Li Hua Experimental Animal Co., Ltd. Blood glucose levels were measured three times (over 16.7 mmol/L) at the 11^th^ week in the Type 2 diabetes model. At the 12^th^ weeks, the rats were randomly collected to detect the urinary albumin/creatinine ratio, which was significantly different from the creatinine ratio of ZDF (FA/+) rats of the same week of age, suggesting that the animal model of DN was successfully established. ZDF (FA/FA) rats were randomized into two groups: the DN group and the UCMSC group. There were five rats in each group. ZDF (FA/+) rats of the same age were used as normal group. In UCMSC group, 1.5 × 10^6^ of UCMSCs were administered via the tail vein at the 12^th^, 14^th^, 15^th^, and 16^th^ weeks. In the DN group and normal group, an equivalent volume of normal saline was administered (Figure 2a). All the rats were sacrificed at the 20^th^ weeks. The serum was collected and stored at −80°C. The kidney was harvested, and kindey tissuese were stored at −80°C for Western blotting, embedded in OCT media for immunofluorescence immediately and fixed in 10% formalin for pathological analyses. All experiments conformed to the Guidelines for Ethical Conductions in the Care and Use of Animals. All animal experiments were conducted in accordance with the permission of the Science and Technology Office of Yunnan Province (Kunming, China) and approved by the Animal Ethics Committee of Yan'an Hospital Affiliated with Kunming Medical University. We made every effort to minimize stress on rats.

#### 2.2.2. Biochemical Determination and Pathological Analysis of Kidney

Serum urea nitrogen and creatinine levels, urinary albumin/creatinine ratio, and blood glucose were measured by a biochemical analyser (BECKMANAU5421, Japan). Paraffin-embedded kidney tissues were cut into 3 *μ*m thick sections, stained with PAS and observed under a microscope. Small pieces of kidney cortex were prefixed with 3.5% glutaraldehyde and then with 1% osmic acid, dehydrated with gradient alcohol and acetone, and then embedded in Araldite 618 (Sigma Aldrich, 10951); ultrathin sections were made and stained with axoyl acetate and lead citrate and then observed with a transmission electron microscope (JEM-1011, Japan).

#### 2.2.3. Immunohistochemical Analysis

The paraffin-embedded renal tissue were cut into 4 *μ*m-thick slices, and the antigens were removed from the hot citrate buffer for 20 min. The antigens were blocked in TBST with 5% BSA for 1 h and then incubated overnight with a primary antibody against TGF-*β*1 (1:100, Cat# A2011, ABclonal) at 4°C. The sections were then incubated with HRP-conjugated secondary antibodies for 30 min, then stained with 3,3⁣′-diaminobenzidine (Vector Laboratories, Burlington, California, United States) and haematoxylin (Nikon Digital Staining DS-Ri1), and observed under a microscope.

#### 2.2.4. Immunofluorescence Analysis

Four micrometer-thick frozen slices were immediately made from kidney tissue and incubated overnight at 4°C in PBS containing rabbit NPHS2 polyclonal antibody (1:200; Proteintech, America). The slices were washed three times with PBS (0.01% Triton X-100) and then incubated at 37°C 1 h with an anti-rabbit IgG antibody (1:1000; Proteintech, America) coupled with Alexa Fluor 594. The cleaning was repeated three times for 5 min each time, and the sections were observed under a fluorescence microscope (Nikon Digital Sight DS-Ri1).

#### 2.2.5. Serum Angptl4 Determination

The expressions of Angptl4 in the serum were detected by an ELISA kit (RDR-Angptl4-Ra, Reddot Biotech).

#### 2.2.6. Western Blot Analysis

Total protein was extracted from −80°C stored kidney tissue, and protein concentration is determined and then separated by SDS-PAGE; transferred to PVDF membranes (Millipore, America); incubated with anti-podocin antibodies (1:1000; Proteintech, America), anti-fibronectin (1:1000; Proteintech, America), anti-Angptl4 (1:2000; Immunoway, America), anti-integrin *β*3 (1:1000; Cell Signalling, America), synaptopodin (1:1000; Proteintech, America), anti-beta actin (1:5000; Proteintech, America), and anti-GAPDH (1:5000; Proteintech, America); incubated with secondary antibodies; and observed with a Super ECL assay (Proteintech, United States).

#### 2.2.7. Cell Experiments

The immortalized rat podocyte cell line was purchased from Beijing Beina Chuanglian Institute of Biotechnology (Beijing, China). Rat podocyte cells were cultured in DMEM supplemented with 10% FBS in 37°C and 5% CO_2_ in an incubator. The cells were seeded in transwell plates and exposed to high glucose (30 mmol/L) with or without UCMSC for 48 h. The glucose concentration in the normal group was 5.5 mmol/L. Cell viability was measured by a CCK-8 assay. The expression levels of the Angptl4, integrin *β*3, and synaptopodin proteins were detected by western blotting. All experiments were performed in triplicate.

### 2.3. Statistical Analysis

Statistical analysis was performed using GraphPad Prism 10.0.2. The measurement data are expressed as the mean ± SD. The differences among multiple groups were compared by one-way ANOVA. *p* < 0.05 was considered to indicate statistical significance.

## 3. Results

### 3.1. UCMSC Therapy Can Improve Renal Function in ZDF (FA/FA) Rats

Compared with the normal group, the DN group showed significant increases in random blood glucose and the urinary albumin–creatinine ratio, indicating that the DN model was successfully established in the 12^th^ week. Compared with those in the DN group, lower levels of serum blood urea nitrogen and serum creatinine were observed in the UCMSC group at the 18^th^ week (*p* < 0.05, Figure 2b) and 20^th^ week (*p* < 0.05, Figure 2c), respectivelly. However, random blood glucose in the DN group was not affected by UCMSC therapy (*p* > 0.05, Figure 2d). UCMSC treatment tended to improve the urinary albumin–creatinine ratio; however, the difference was not significant (*p* > 0.05, Figure 2e).

### 3.2. UCMSC Therapy Improves Kidney Pathological Injury and Podocyte Injury in ZDF Rats

**Figure 3:**
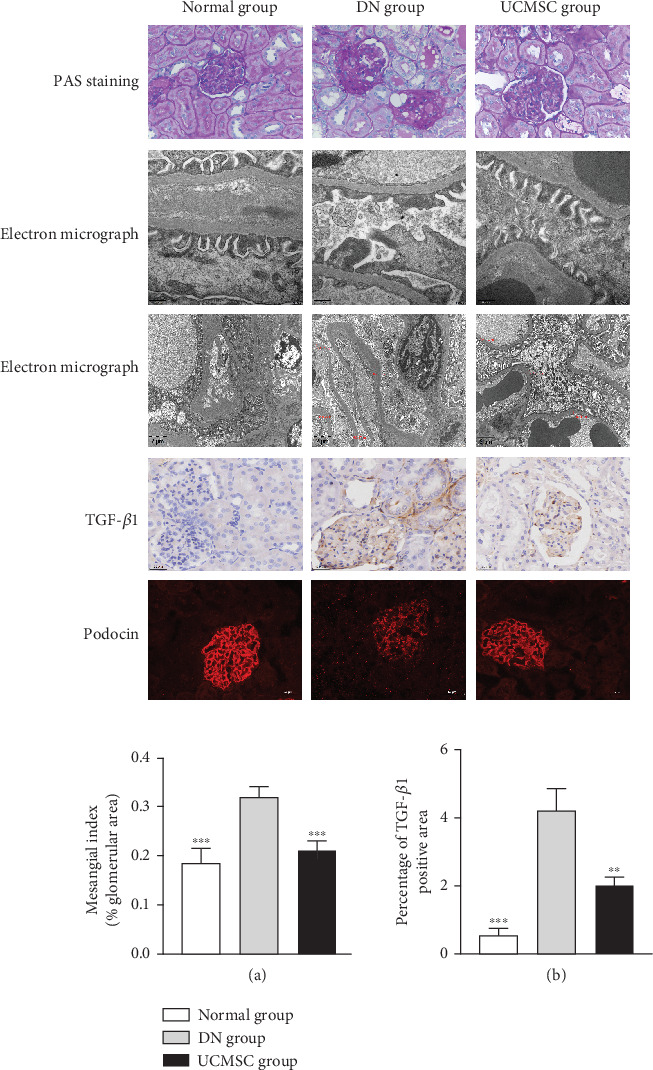
Pathological staining (×200) and transmission electron microscope observation (×20,000, ×5000) of kidney tissue. TGF-*β*1 immunohistochemistry (×400) and podocin immunofluorescence (×200) of kidney tissue. (a) Statistics of extracellular matrix deposition and fibrosis degree in PAS staining. (b) Statistical chart of TGF-*β*1 positive area percentage versus DN group. ∗*p* < 0.05, ∗∗*p* < 0.01, ∗∗∗*p* < 0.001.

According to PAS staining, the DN group showed severer proliferation of the mesangial matrix in the glomeruli than the normal group (*p* < 0.05, Figure 3a). Under electron microscopy, obvious foot process fusion and a thicker glomerular basement membrane were observed in the DN group. Kidney pathological changes in the DN group indicated that the DN model was successfully established. UCSMC treatment can significantly alleviate the proliferation of the mesangial matrix in the glomeruli in DN rats (*p* < 0.05, Figure 3a). Electron microscopy suggested that UCMSC improved foot process fusion and the thickness of the basement membrane. Compared to the normal group, TGF-*β*1 in kidney tissue was significantly increased in the DN group (*p* < 0.05, Figure 3b), while UCMSC could significantly downregulate the expression TGF-*β*1 (*p* < 0.05, Figure 3b), which showed that UCMSC could attenuate renal pathological injury in DN rats. Immunofluorescence indicated that the expression of podocin in the DN group was significantly inhibited, while the expression of podocin in the UCMSC group was restored, suggesting that UCMSC could improve podocyte damage in ZDF rats (Figure 3).

### 3.3. The UCMSC Ameliorates Kidney Injury in ZDF Rats Through Inhibiting Angpltl4 in Glomerulus

**Figure 4:**
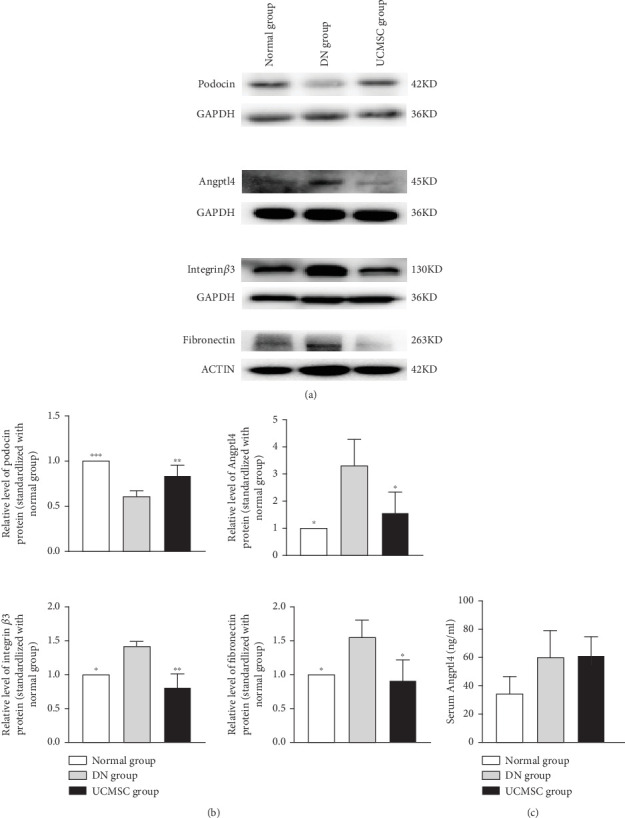
Protein expression of podocin, Angptl4, integrin *β*3, and fibronectin in kidney tissue. (a) The levels of podocin, Angptl4, integrin *β*3, and fibronectin were examined by Western blotting. (b) Statistical plots comparing the DN group. ∗*p* < 0.05, ∗∗*p* < 0.01, ∗∗∗*p* < 0.001. (c) The expression of serum Angptl4 by ELISA analysis.

To elucidate the mechanism by which UCMSC improve podocyte injury in DN rats, we analysed the levels of podocin, Angptl4, integrin *β*3, and fibronectin in renal tissue by western blot. Podocin expression was significantly inhibited in the DN group compared with the normal group (*p* < 0.05), and the expression of Angptl4, integrin *β*3, and fibronectin was significantly increased (*p* < 0.05), while podocin expression was significantly restored by UCMSC treatment (*p* < 0.05), and the expression of Angptl4, integrin *β*3, and fibronectin were significantly inhibited by UCMSC treatment (*p* < 0.05). These results suggest that UCMSC may ameliorate kidney injury in ZDF rats by inhibiting Angpltl4/integrin *β*3 pathway in the glomerulus (Figure 4a,b). We measured Angptl4 concentration in serum by ELISA and found that UCMSC had no effect on serum Angptl4 (Figure 4c).

### 3.4. UCMSC Improved Podocyte Injury Under High-Glucose Conditions Through the Angptl4/Integrin *β*3 Pathway In Vitro

**Figure 5:**
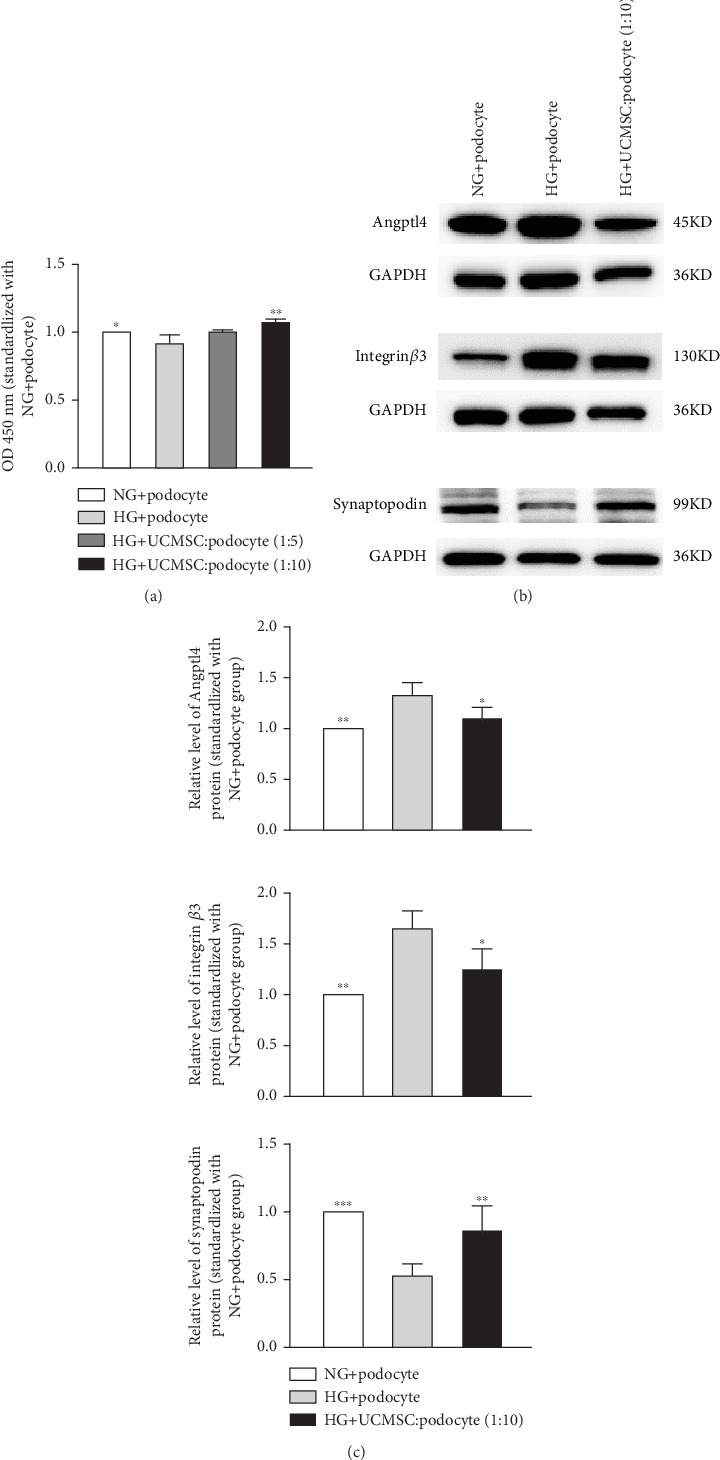
(a) Restoration of podocyte activity under conditions of high glucose stimulation by UCMSC treatment versus HG + podocyte, ∗*p* < 0.05, ∗∗*p* < 0.01. (b) The expression of Angptl4, integrin *β*3, and synaptopodin in podocytes was examined by Western blot analysis. (c) Statistical plots compared with the HG + podocyte group. ∗*p* < 0.05, ∗∗*p* < 0.01, ∗∗∗*p* < 0.001.

Podocytes were stimulated with high-glucose conditions (30 mmol/L) with or without UCMSC, and CCK-8 assay showed that podocyte activity was significantly decreased under high-glucose conditions, while UCMSC coculture restored podocyte activity (*p* < 0.05) (Figure 5a). Western blot analysis revealed significantly increased expression of Angptl4 and integrin *β*3 in podocytes subjected to high-glucose stimulation (*p* < 0.05) and can be reversed by coculture with UCMSC (*p* < 0.05). The expression of synaptopodin decreased in podocytes after high glucose stimulation but was restored by coculture with UCMSC (*p* < 0.05) (Figure 5b,c).

## 4. Discussion

To date, DN is still incurable and is a major clinical challenge. Studies have shown that MSCs have the potential to treat DN. However, the underlying mechanism has not been elucidated. Most studies have used the STZ-induced DN model, which is characterized by mild renal damage [24]. In this study, spontaneous Type 2 diabetes rat line ZDF (FA/FA) was used as the DN model, and the pathological damage to the kidney in this model was more similar to that in human DN [22]. After UCMSC treatment, the renal function and pathological injury of ZDF rats were significantly improved. In addition, the expression of fibronectin, one of the main components of the extracellular matrix (ECM), was significantly inhibited after UCMSC treatment. Early intervention with mesenchymal stem cells prevents nephropathy in diabetic rats by ameliorating the inflammatory microenvironment [25, 26]. The expression of transforming growth factor-*β*1 (TGF-*β*1) can lead to abnormal ECM production, resulting in glomerular sclerosis and interstitial tubule injury [27]. Our study revealed that the expression of TGF-*β*1 in kidney tissue was decreased after UCMSC treatment. No significant effect on blood glucose after UCMSC treatment was found in this study. This finding is inconsistent with the results of the STZ-induced DN model, possibly because different animal models were used, but it also suggests that MSCs improve DN not by lowering blood glucose. Proteinuria in rats tended to decrease during UCMSC treatment, but the difference was not significant. However, electron microscopy and immunofluorescence analysis of podocin revealed that UCMSC treatment improved podocyte injury in the DN model. Some scholars have also found shown that MSCs can improve the podocyte damage in a DN model [28, 29]. However, the mechanism underlying the protection of podocytes has not been elucidated.

In recent years, studies have shown that increased Angptl4 secreted by podocytes is an important pathogenic factor leading to podocyte injury in kidney diseases, especially minimal change disease and DN. There are two types of Angptl4 proteins in the body, circulating Angptl4 and podocytes secreting Angptl4. Circulating Angptl4 was mainly expressed in liver and adipose cells and secreted into circulation to regulate plasma lipid metabolism. We found that UCMSC treatment did not affect circulating Angptl4 in DN rats. However, Angptl4 in kidney tissues is mainly secreted by podocytes in DN and plays important role locally in the kidney. The homing of mesenchymal stem cells was verified using positron emission tomography (PET) imaging, and some scholars have found that MSCs can target damaged kidneys [30]. UCMSC entered the injured kidney mainly through paracrine function to repair renal injury [31]. Our study revealed that the expression of Angptl4 in kidney tissue of DN rats was increased and that the expression of Angptl4 in renal tissue was significantly inhibited after UCMSC treatment. In addition, UCMSC treatment significantly decreased the expression of Angptl4 in high-glucose-stimulated podocytes in vitro. These results have not been reported before and deserves further study.

Foot disappearance is a dynamic, high-energy, and complex process based on signal transduction, and the rearrangement of actin cytoskeleton is closely related to the dynamics of integrin adhesion [32]. Synaptopodin is distributed in the foot process of podocytes [33]. We use STRING and Hitpredict to predict the interaction protein of Angptl4. It is found that Angptl4 interacts with the integrin family, and integrins are closely related to podocyte damage. Integrins are key molecules that mediate cell adhesion and participate in podocyte migration [19, 34]. Studies have shown that the integrin *β*3 subtype is expressed on podocytes. Activation of integrin *β*3 has been shown to lead to loss of the foot process and proteinuria by activating downstream intracellular signal molecules, such as the RhoA and YAP [35, 36]. Inhibition of integrin *β*3 with anti-integrin *β*3 antibodies or small molecule inhibitors can reduce proteinuria [37, 38]. The interaction between Angptl4 and integrin *β*3 has been verified in hypoxia-induced vascular permeability model [21]. However, the mechanism of Angptl4 and integrin *β*3 in podocytes injury in DN has not been reported. To further verify the role of Angptl4 in DN, we detected integrin *β*3 in podocytes under high glucose conditions. We found that integrin *β*3 was significantly inhibited in podocytes after UCMSC treatment and that the xpression of synaptopodin was restored. We also detected the expression of integrin *β*3 in renal tissue and found that the expression of integrin *β*3 in renal tissue was significantly upregulated, while the expression of integrin *β*3 was inhibited after UCMSC treatment. These results suggest that UCMSC may restore podocyte skeletal protein expression by inhibiting podocyte Angptl4 and integrin *β*3.

In our study, we found that UCMSC protected against podocyte damage in a DN model by inhibiting the Angptl4/integrin *β*3 pathway. Our study identified a new mechanism of UCMSC-mediated podocyte protection, and targeting podocyte damage induced by Angptl4/integrin *β*3 pathway is a promising approach for treating DN. In addition, how UCMSC inhibits Angptl4 expression in podocytes is still worthy of further study, which will make a foundation for modifying UCMSC to improve its therapeutic effect on DN in the future.

## 5. Conclusions

**Figure 6:**
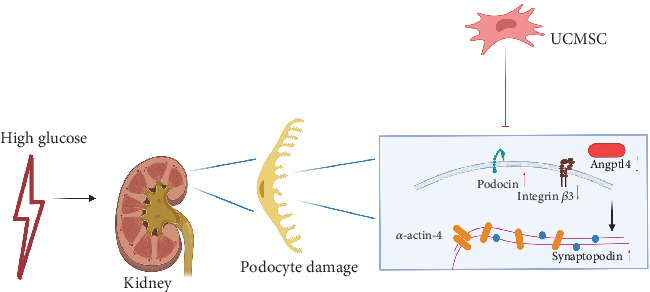
Mechanism diagram (created by BioRender).

UCMSC therapy improved renal function and pathological changes in renal tissues in DN ZDF (FA/FA) rats. In addition, the ability of UCMSC therapy to alleviate podocyte injury in the DN model may be related to the inhibition of the Angptl4/integrin *β*3 pathway (Figure 6).

## Data Availability

The datasets used and analyzed during the current study are available from the corresponding authors upon reasonable request.
